# Monitoring temozolomide treatment of low-grade glioma with proton magnetic resonance spectroscopy

**DOI:** 10.1038/sj.bjc.6601593

**Published:** 2004-02-17

**Authors:** P S Murphy, L Viviers, C Abson, I J Rowland, M Brada, M O Leach, A S K Dzik-Jurasz

**Affiliations:** 1 Cancer Research UK Clinical Magnetic Resonance Research Group, The Institute of Cancer Research and The Royal Marsden NHS Trust, Sutton, Surrey SM2 5PT, UK; 2 Academic Department of Neuro-oncology, The Institute of Cancer Research and The Royal Marsden NHS Trust, Sutton, Surrey SM2 5PT, UK

**Keywords:** magnetic resonance spectroscopy, low-grade glioma, temozolomide, treatment response

## Abstract

Assessment of low-grade glioma treatment response remains as much of a challenge as the treatment itself. Proton magnetic resonance spectroscopy (^1^H-MRS) and imaging were incorporated into a study of patients receiving temozolomide therapy for low-grade glioma in order to evaluate and monitor tumour metabolite and volume changes during treatment. Patients (*n*=12) received oral temozolomide (200 mg m^−2^ day^−1^) over 5 days on a 28-day cycle for 12 cycles. Response assessment included baseline and three-monthly magnetic resonance imaging studies (pretreatment, 3, 6, 9 and 12 months) assessing the tumour size. Short (TE (echo time)=20 ms) and long (TE=135 ms) echo time single voxel spectroscopy was performed in parallel to determine metabolite profiles. The mean tumour volume change at the end of treatment was −33% (s.d.=20). The dominant metabolite in long echo time spectra was choline. At 12 months, a significant reduction in the mean choline signal was observed compared with the pretreatment (*P*=0.035) and 3-month scan (*P*=0.021). The reduction in the tumour choline/water signal paralleled tumour volume change and may reflect the therapeutic effect of temozolomide.

Low-grade gliomas (World Health Organisation (WHO) grade II astrocytoma, oligodendroglioma and mixed oligoastrocytoma) tend to be slow-growing tumours (Behin *et al*, 2003) and there remains considerable debate about the appropriate first-line treatment (Stieber, 2001; Rees, 2002). A policy of surveillance is widely accepted and active treatment in the form of surgery or radiotherapy is generally offered at the time of tumour progression. With the recognition of chemoresponsiveness of grade III anaplastic oligodendroglioma (Cairncross *et al*, 1994) and a suggestion of efficacy of chemotherapy in grade II oligodendroglioma (Glass *et al*, 1992; Mason *et al*, 1996; Peterson *et al*, 1996), an increasing number of patients with grade II neuroepithelial tumours have been treated with chemotherapy. However, this approach has largely been reserved for patients who failed first-line treatment with surgery and radiotherapy. Initial reports of the effectiveness of chemotherapy in grade II glial tumours employed nitrosoureas usually as the PCV (procarbazine, CCNU (1-(2-chloroethyl)-3-cyclohexyl-1-nitrosourea), vincristine) regimen (Glass *et al*, 1992; Mason *et al*, 1996; Peterson *et al*, 1996).

Temozolomide is a well-tolerated oral alkylating agent with almost complete bioavailability that crosses the intact blood–brain barrier. It is licensed for the treatment of recurrent malignant glioma (glioblastoma multiforme and anaplastic astrocytoma) (Yung *et al*, 1999, 2000; Brada *et al*, 2001). The 35% response rate in WHO grade III astrocytoma (anaplastic astrocytoma) (Yung *et al*, 1999) in comparison to 6–8% response rate in grade IV tumours (glioblastoma multiforme) (Yung *et al*, 2000; Brada *et al*, 2001) suggests a possible trend of increasing efficacy in lower grade tumours previously noted for nitrosourea-containing chemotherapy (Rajan *et al*, 1994). On the basis of excellent tolerance and potential efficacy of temozolomide in lower grade tumours, we evaluated temozolomide as the primary treatment following surgery in patients with grade II glial tumours in a single arm phase II study. The results of the study are the subject of a separate report (Brada *et al*, 2003).

Imaging with either MRI or CT remains the mainstay for radiological response assessment in low-grade glioma management (Ricci and Dungan, 2001). Serial measurement of either the low-density regions on CT or those areas of high signal intensity relative to normal adjacent brain on T2-weighted (T2W) MR images and FLAIR (FLuid Attenuated Inversion Recovery) are used to quantify treatment response. Despite advances in imaging, delineation of low-grade tumours is difficult. These lesions seldom enhance following administration of paramagnetic contrast agents due to an intact blood–brain barrier, and the diffusely infiltrative nature of these tumours makes the assessment of tumour boundaries difficult.

Proton magnetic resonance spectroscopy (^1^H-MRS) is a noninvasive technique describing aspects of tumour biology that can be readily incorporated into standard diagnostic MRI protocols (Smith and Stewart, 2002). The potential diagnostic and prognostic capabilities of ^1^H-MRS in brain tumours have received considerable attention (Tedeschi *et al*, 1997; Nelson *et al*, 1999; Poptani *et al*, 1999; Preul *et al*, 2000). It would be reasonable to expect that morphological and clinical responses are preceded by a change in the tumour biochemistry. It remains to be answered whether such changes are detectable by MRS and if so whether they provide sensitive indices of response and/or prognosis.

The aim of this study therefore was to assess the serial metabolic changes detectable by ^1^H-MRS in low-grade glioma patients receiving single-agent temozolomide therapy.

## MATERIALS AND METHODS

### Patient selection

In all, 13 patients with histologically verified WHO grade II glioma (nine astrocytoma, four oligodendroglioma) were recruited. They had evaluable disease on imaging and no previous antitumour treatment other than surgery (biopsy or debulking surgery), with either stable or progressive disease. The median age was 39 years; seven were men and six were women. Patients with radiological or histological evidence of transformation to high-grade tumours were excluded. Patients in need of urgent decompressive surgery or radiotherapy due to rapidly evolving neurological deficit and patients who had raised intracranial pressure and were corticosteroid dependent were not included. Treatment commenced 1–44 months (median 22 months) after histologically confirmed diagnosis.

The local Scientific and Ethics Committees approved the study protocol, and patients were required to sign informed consent prior to entry into the study.

### Treatment

Patients received temozolomide at 200 mg m^−2^ day^−1^ for 5 consecutive days on a 28-day cycle and the dose and frequency were adjusted according to standard toxicity criteria (Brada *et al*, 1999, 2001; Yung *et al*, 1999). Patients were intended to receive a maximum of 12 cycles of temozolomide; treatment was discontinued in the presence of disease progression or due to unacceptable toxicity.

### Magnetic resonance imaging

All MR measurements were performed on a 1.5 T. Siemens Vision (Erlangen, Germany) system using the manufacturer's circularly polarised head coil and the same protocol was used for each visit throughout the trial. The full MRI protocol consisted of standard diagnostic MRI together with MRS acquisitions. Precontrast, the images were acquired using: T1-weighted (T1W) coronal (TR (repetition time)=570 ms, TE (echo time)=14 ms, slice thickness=8 mm), T2W coronal (TR=4500 ms, TE=99 ms, slice thickness=8 mm) and axial FLAIR (TR=8000 ms, TE=119 ms, TI (inversion time)=2400 ms, slice thickness=6 mm, slice separation=0 mm). All patients received bolus administration of double-dose (0.2 mmol kg^−1^) Gd-DTPA (gadolinium diethylenetriamine pentaacetic acid, Schering Healthcare, UK). Postcontrast T1W coronal scans were acquired with the same sequence parameters as the precontrast acquisition. The study was performed within 2 weeks before the start of treatment and three monthly thereafter during the 12 months of treatment, at 3, 6, 9 and 12 months.

Tumour size was assessed as the region of high signal intensity on FLAIR images by two observers (LV, CA) in consensus. The FLAIR sequence was chosen for quantification because it is standard practice at our institution, and of the sequences available to us, it is the sequence most sensitive to tumour-induced changes. The assessment was not blinded. The abnormality was outlined manually on each two-dimensional slice and the area calculated via an automated procedure. The volume of the FLAIR abnormality was calculated by summing all the areas and multiplying by the thickness of all the involved slices. Standard response criteria were applied, where partial response is defined as >50% reduction in size and disease progression is >25% increase in tumour size. For purposes of this study, a 25–50% reduction in tumour size has been considered as a minimal response. A change in size between that of a minimal response and that of disease progression was classified as stable disease.

### Magnetic resonance spectroscopy

All spectra were acquired precontrast, using single voxel STEAM (Stimulated Echo Acquisition Mode) methodology with a mean voxel size of 10.9 cm^3^ (range: 10.6–12.2 cm^3^). The criteria used by an experienced radiologist (ADJ) for positioning the spectroscopic voxel included: (1) voxel placement centrally within the FLAIR-defined abnormality, including where evident, a distinct mass lesion (as compared with white matter tract abnormality). Substantial radiologically defined cystic regions were excluded from the voxel. (2) Exclusion from the voxel of brain tissue deemed normal on the FLAIR image. For follow-up spectroscopy, images detailing the previous voxel position were available to the radiologist ensuring voxel colocation between studies. Voxel placement at subsequent examinations was performed by the same radiologist. Spectra were acquired at long (TE=135 ms) and short (TE=20 ms) echo times with TR=2500 ms and number of averages=64. A water reference spectrum was acquired from the same voxel with TE=20 ms, TR=2500 ms and number of averages=4. Spectral analysis was performed using the VARPRO (Variable Projection) method (van den Boogaart *et al*, 1994) with MRUI (Magnetic Resonance User Interface) software. Both metabolite and water peaks (from the reference spectrum) were fitted using Lorentzian line shapes. The metabolite signal areas were normalised to the relevant water signal area (TE=20 ms). For choline and creatine quantification, the peak areas of the long echo time spectra were determined and divided by the short echo time water spectrum. For *N*-acetyl aspartate (NAA), the short echo time spectra were quantified due to the very low signal-to-noise of this peak at long echo time. The short echo time spectra also served to detect lipid methyl and methylene signals.

### Statistics

Normality within a data set was tested using a Shapiro–Wilk test and paired groups of data were compared using a paired *t*-test. Differences in the mean of the choline/water values were assessed between pretreatment values and the data at 3, 6, 9 and 12 months.

## RESULTS

A summary of data for all patients receiving temozolomide for the full 12-month course of treatment is given in Table 1

**Table 1:** Summary of imaging and spectroscopy results for the 12 patients completing 12 months of treatment

		**Magnetic resonance spectroscopy**			
		**Pretreatment**		**Post-treatment**	
**Patient**	**Magnetic resonance imaging Volume change (12months) (%)**	**Choline/water (× 10^−5^) (TE=135 s)**	**NAA/water (× 10^−5^) (TE=20 ms)**	**% choline at 12 months (TE=135 ms)**	**% NAA at 12 months (TE=20 ms)**
1	+12	12	19	−16	−14
2	−34	15	19	−100	−100
3	−32	27	0	−25	No NAA
4	−39	13	0	—a	—a
5	−40	27	6	−35	+160
6	−38	26	0	−60	No NAA
7	−51	22	19	+14	−25
8	−47	17	22	−42	−40
9	−52	26	12	+24	+220
10	+3	21	0	+10	No NAA
11	−44	17	22	−50	+328
12	−32	22	0	−51	No NAA
					
Mean (s.d.)	−33 (20)	20 (6)	10 (10)	−30 (37)	76 (160)
					

a The instances where the full spectroscopy examination could not be completed are indicated. The volume column refers to the % change as determined by FLAIR MRI. The spectroscopic quantification of choline is that derived from the long echo (TE=135 ms) time acquisitions. The quantification of NAA is derived from the short echo time spectra (TE=20 ms). NAA=*N*-acetyl aspartate. TE=Echo time.

.

### Clinical summary

Out of 13 patients who started treatment, 12 completed the 12-month cycle. One patient was removed from the study after 9 months due to deterioration in physical condition and was radiologically confirmed to have undergone high-grade transformation. The following results apply to the 12 patients who completed the full course of treatment.

### Imaging analysis

**Figure 1:**
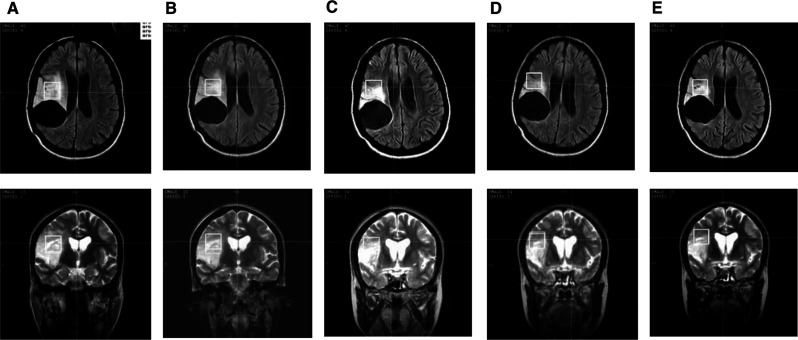
A series of images from patient #5 demonstrating the reduction in tumour volume apparent on FLAIR (top) and T2W-FSE (bottom) images. Images displayed are from (**A**) pretreatment and post-treatment at (**B**) 3 months, (**C**) 6 months, (**D**) 9 months and (**E**) 12 months. Note the visible reduction in abnormal signal intensity on the FLAIR image during the course of the patient's treatment with temozolomide. A postsurgical cavity (seen as low signal in the axial images) is situated immediately posterior to the abnormal high signal intensity. (The cross hairs in each image indicate the magnetic isocentre. Where the voxel is not represented as a single square was due to the obliquity of the underlying reference images).

A summary of the imaging data is provided in Table 1. All patients had an initial reduction in tumour volume as measured at 3 and 6 months. The mean reduction was 23% (range −4% → −47%) at 6 months and 33% (range +12% → −52%) at 12 months. Two patients showed a small increase in tumour size at 12 months. An example of tumour volume reduction is shown in Figure 1

. Therefore 12 months following the start of treatment, two patients had stable disease, eight had a minimal response and two had a partial response.

### Spectroscopy

Spectroscopy was successfully completed on all patients at 3 months, 10 out of 12 patients at 6 months, nine out of 12 patients at 9 months and 11 out of 12 patients at 12 months. Spectroscopy was not performed on six occasions due to technical problems or the patients being unable to tolerate the full MR protocol.

**Figure 2:**
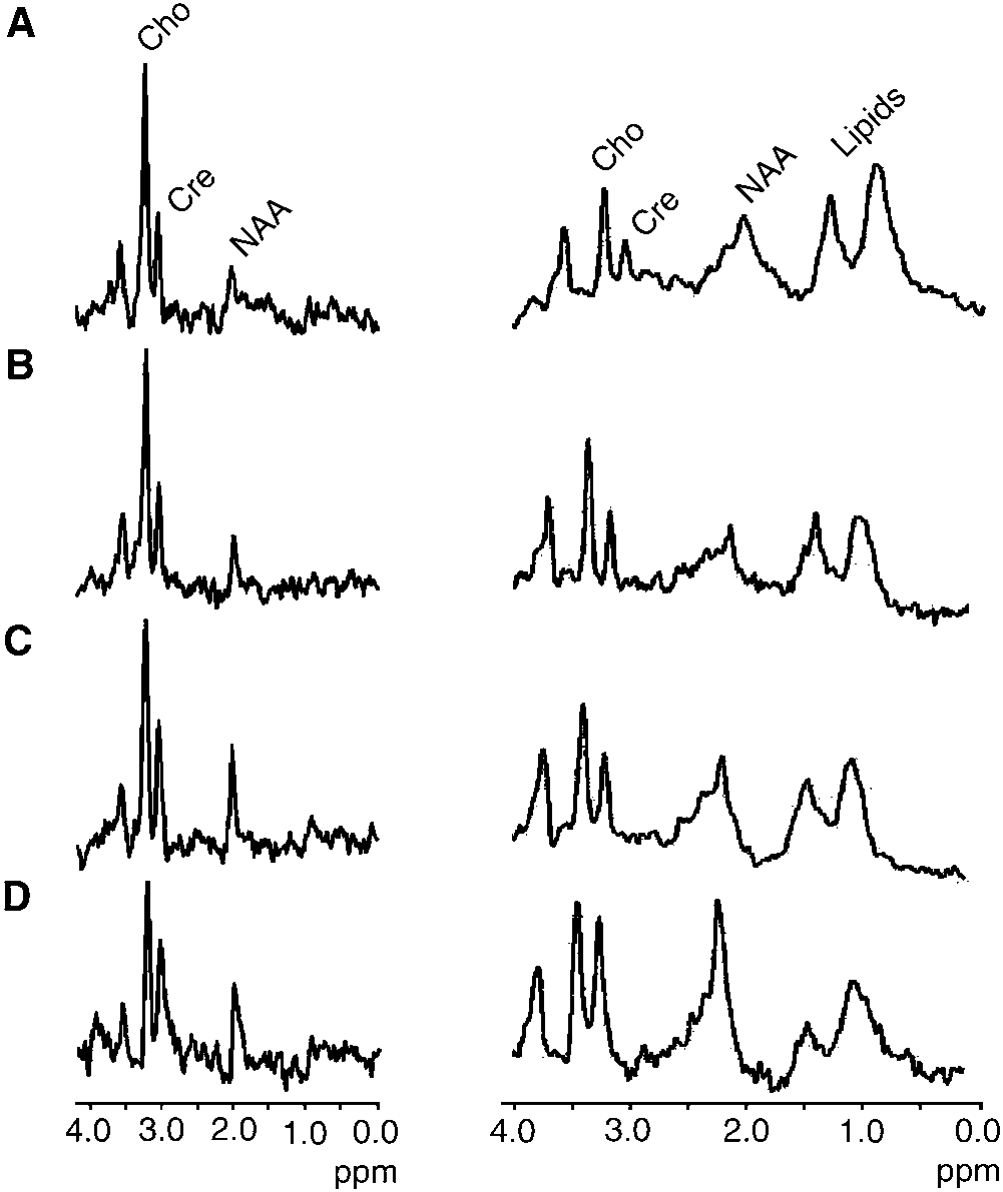
Long echo time (TE=135 ms, left of figure) and short echo time (TE=20 ms, right of figure) STEAM spectra obtained from patient #5 during temozolomide treatment: (**A**) pretreatment, (**B**) at 3 months, (**C**) at 6 months and (**D**) 9 months. Within both series a progressive decrease in the choline/creatine ratio is observed, suggesting a reduced membrane metabolism and diminishing cellular density. Also note the increasing conspicuity of the NAA peak. *N*-acetyl aspartate is a specific neuronal marker and may reflect the regression of tumoral tissue and repopulation of normal brain matter.

An example series of long echo time spectra (TE=135 ms) obtained during temozolomide treatment is shown in Figure 2

(left of figure). These spectra are from patient #5 whose tumour volume had reduced by 40% at 12 months. The MRS voxel positions are shown in the FLAIR and T2W images in Figure 1. The principal ^1^H-MRS features of a low-grade glioma are illustrated in the pretreatment spectrum in Figure 2 (A, left of figure): a dominant choline signal, low-intensity creatine and NAA and no quantifiable lipid or lactate.

Short echo time spectra (TE=20 ms) from the same patient are also shown in Figure 2 (right of figure). The signal-to-noise ratio is considerably greater than at longer echo time for the same number of acquisitions, indicating short metabolite T2 values. The lipid methyl (0.9 ppm) and methylene (1.2 ppm) peaks not visible at TE=135 ms display high signal intensity.

The choline data displayed in Table 1 are those obtained from the analysis of the long echo time (TE=135 ms) spectra. For quantitative temporal analysis, the only consistently fitted peak in all patients was choline (with the exception of patient #4). The intensities of NAA and creatine were commonly too low for quantification at long echo time.

At 6 months, seven out of 10 patients demonstrated a reduction in choline signal. At 12 months, eight out of 11 patients showed choline reduction. One patient (#2) demonstrated a complete loss of choline signal at 6 months that persisted in subsequent scans.

*N*-acetyl aspartate was quantifiable in seven out of 12 patients in the short echo time pretreatment spectra. Three patients demonstrated large increases in NAA concentration (+160%, +220% and +328%) at 12 months. Pretreatment lipid was only observed in three of the 12 patients in the short-echo time spectra (patients #2, #5 and #9).

Figure 3

**Figure 3:**
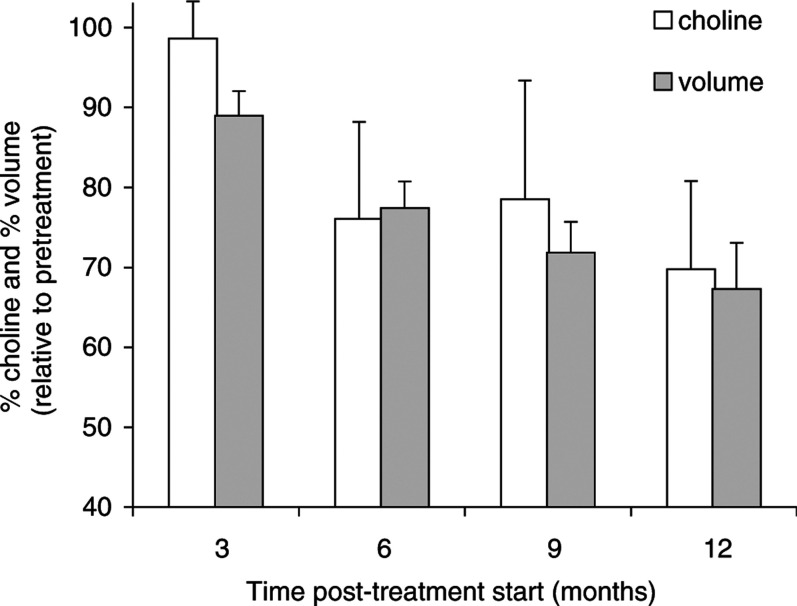
Mean choline and mean volume expressed as a percentage for all 12 patients completing the 12 months of treatment. The % choline is calculated by normalising the TE=135 ms signal to water. For each patient all post-treatment values were normalised to the pretreatment volume and choline data. The error bars represent the s.e. associated with the mean. Note the almost parallel trend in the reduction of tumour choline and volume.

shows the mean percentage of choline and FLAIR tumour volume from the 12 patients. For the purpose of illustration, all post-treatment values have been normalised to the pretreatment choline and volume data, respectively.

### Statistics

The Shapiro–Wilk test indicated that there was no statistically significant evidence to assume that the choline/water data were not normally distributed at each of the experimental time points, and therefore paired *t*-tests were performed between paired groups of data. There was a statistically significant difference between the pretreatment and the 12-month choline/water data (*P*=0.035) and the 3- and 12-month data (*P*=0.021). No difference was detected between the pretreatment and 3-month data (*P*=0.73). No statistically significant differences were detected between the other groups of data. Statistical significance was taken at the 95% level.

## DISCUSSION

Morphological imaging often does not adequately reflect underlying tumour biology and is illustrated by low-grade gliomas where the tumour periphery is often seemingly well circumscribed but histologically quite the opposite (Behin *et al*, 2003). There is therefore a considerable incentive to develop alternative measures of therapeutic response in humans. The results of this study suggest that therapeutically induced metabolic changes are clinically detectable using ^1^H-MRS.

### Metabolic changes

#### Choline

The decrease in tumour volume with temozolomide therapy was closely matched by a decrease in the choline/water ratio during treatment. The potential biological and clinical significance of this observation is reinforced by the statistically significant higher level of choline/water pretreatment and at 3 months as compared with the end treatment values.

It is recognised that MRS-determined choline reflects membrane synthesis (Tedeschi *et al*, 1997). Furthermore, the choline resonance has been correlated with cell density and with indices of cellular proliferation (Miller *et al*, 1996). The detected decrease in choline could be a consequence of the therapeutic effect of temozolomide and subsequent neoplastic cell death. An increase in the choline/water suggests an increase in membrane metabolism and cell density, suggesting an absence or loss of therapeutic response. In high-grade glioma, choline signals are predominantly elevated at the tumour periphery where there is enhanced cellular proliferation supported by an adequate blood supply. In the core of a high-grade tumour, however, there is a low choline signal as the viable cell count is reduced due to necrosis. Necrosis is not a feature of low-grade glioma and therefore, in the absence of high-grade transformation treatment-mediated cell death is a more likely cause of choline reduction. A reduction in choline signal intensity within the context of this study may be a metabolic indicator of response to temozolomide therapy.

Furthermore, a reduction in the tumour choline does not follow the previously reported metabolic evolution of this tumour type. In a study (Tedeschi *et al*, 1997), serial measurements of the choline signal were carried out in patients with low-grade astrocytoma (WHO grade II), low-grade oligodendroglioma (WHO grade II), anaplastic astrocytomas (WHO grade III) and high-grade astrocytomas (WHO grade IV). This study categorised patients radiologically into stable or progressive disease. Patients with stable disease showed tumour choline signal changes between −33 and +28% with a mean of 0.4%. Those individuals with progressive disease demonstrated choline signal changes in the range +46 to +104% with a mean of 55.6%. In this current study, the reduction in choline demonstrates changes opposite to those associated with progressive disease and remains distinct from the metabolically static situation of stable disease.

#### *N*-acetyl aspartate

*N*-acetyl aspartate was visible in seven out of 12 patients in the short echo time pretreatment spectra. Three patients demonstrated large increases in NAA concentration (160%, 220% and 328%], all of whom demonstrated a response: two minimal and one partial. The images from one of these patients (160% NAA) are used as an example in Figure 1. It can be seen from the overlaid MRS voxel that during treatment the amount of radiologically normal tissue within the voxel increases as the tumour reduces in size. This is supported by the increase in NAA with time as the normal tissue returns to the site originally infiltrated by tumour tissue. This demonstrates a metabolic feature that could be explained by the diffusely infiltrative nature of low-grade glioma, resulting in invasion rather than destruction of surrounding brain tissue. If tumour cells are therapeutically killed, the normal tissue may return into the space once occupied by tumour. As NAA is a sensitive indicator of neuronal density (Nadler and Cooper, 1972), the increase in NAA supports the proposed mechanism. Sensitivity to this process will, however, be technology dependent. If the voxel is sited in the centre of a large tumour then it may require a large change in tumour volume to detect an alteration in NAA. This may reflect why NAA did not increase in some tumours that demonstrated a substantial reduction in tumour volume.

It is also possible that signal processing issues may have influenced NAA quantification. The complex of peaks around 2.0 ppm often poses difficulties in automated fitting routines. The inevitable minor variation in voxel positioning in serial examinations could have resulted in some variation in the degree to which normal brain NAA influences the spectrum. A spectroscopic imaging approach would address these issues.

#### Lipid

No methyl or methylene lipid signals were observed in the long echo time spectra, before or during treatment. Since MRS- visible mobile lipids are associated with necrosis in brain tumours (Barba *et al*, 1999) this observation is consistent, acknowledging the limits in sensitivity of MR with the histology of low-grade tumours. At short echo time, however, low levels of lipid methylene and methyl signals were detected in three out of 12 patients. These three patients demonstrated improvement in clinical symptoms and MRI volume reduction. The lipid signal in Figure 2 (right of figure) reduces during treatment. However, as only three patients displayed quantifiable lipids, it was inappropriate to calculate the statistical significance of these changes with respect to treatment response. As the majority of individuals showing clinical improvement did not express any significant spectroscopically detectable lipid resonance, its presence does not appear to be prognostic.

#### Other metabolites

Metabolites other than choline, NAA or lipid were generally of too low a concentration to be measured in a serial manner. In those patients in whom creatine could be measured in sufficient concentration, the creatine/water signal showed no significant change during treatment.

Methodologically, the application of a single voxel approach to studying these tumours may cause ambiguity. The heterogeneity of metabolic response cannot be assessed with a single voxel approach. Also, it is not readily feasible (with NAA, for example) to discriminate between tumour cells dying and normal tissues returning within the spectroscopic voxel. The result is likely to be a combination of both phenomena, particularly with small tumours where post-treatment normal tissue can be seen radiologically within the voxel. However, many of these low-grade gliomas were sufficiently large to give confidence that radiologically defined normal tissue was not within the voxel even after tumour shrinkage. Another important methodological issue is the study-to-study placement of the voxel. Some of the changes between the spectra could be due to study-to-study misregistration. However, it is unlikely that misregistration will result in signal change in a particular direction and so the introduced error is likely to be an overall uncertainty rather than a bias.

## CONCLUSION

Our results indicate that changes in the ^1^H-MRS profile of low-grade glioma that parallel volume changes in the tumour can be detected during the course of temozolomide treatment. Furthermore, our study indicates that metabolic changes do not apparently precede changes in tumour volume within the limits of the measurement sensitivity and temporal sampling. Early metabolic response may be a heterogeneous phenomenon below the limits of sensitivity of the current technique. Methodological improvements, including higher field strength and a spectroscopic imaging approach are likely to enhance the value of investigating associations between metabolic response and the morphological consequence of chemotherapy.
